# Magnetic Phase-Change Microcapsules with High Encapsulation Efficiency, Enhancement of Infrared Stealth, and Thermal Stability

**DOI:** 10.3390/ma17194778

**Published:** 2024-09-28

**Authors:** Chun-Wei Chang, Zheng-Ting Chen, Yeng-Fong Shih

**Affiliations:** Department of Applied Chemistry, Chaoyang University of Technology, Taichung 413310, Taiwan; adad94033@gmail.com (C.-W.C.); a87012155@gmail.com (Z.-T.C.)

**Keywords:** phase-change microcapsules, magnetic additive, ferric ferrous oxide, infrared emissivity

## Abstract

Due to energy shortages and the greenhouse effect, the efficient use of energy through phase-change materials (PCMs) is gaining increased attention. In this study, magnetic phase-change microcapsules (Mag-mc) were prepared by suspension polymerization. The shell layer of the microcapsules was formed by copolymerizing methyl methacrylate and triethoxyethylene silane, with the latter enhancing the compatibility of the shell layer with the magnetic additive. Ferric ferrous oxide modified by oleic acid (Fe_3_O_4_(_m_)) was added as the magnetic additive. Differential scanning calorimetry (DSC) testing revealed that the content of phase-change materials in microcapsules without and with ferric ferrous oxide were 79.77% and 96.63%, respectively, demonstrating that the addition of Fe_3_O_4_(_m_) improved the encapsulation efficiency and enhanced the energy storage ability of the microcapsules. Laser particle size analysis showed that the overall average particle sizes for the microcapsules without and with ferric ferrous oxide were 3.48 μm and 2.09 μm, respectively, indicating that the incorporation of magnetic materials reduced the size and distribution of the microcapsules. Thermogravimetric analysis indicated that the thermal stability of the microcapsules was enhanced by the addition of Fe_3_O_4_(_m_). Moreover, the infrared emissivity of the microcapsule-containing film decreased from 0.77 to 0.72 with the addition of Fe_3_O_4_(_m_) to the shell of microcapsules.

## 1. Introduction

With the advancement of technology, the demand for various resources is increasing steadily, along with a gradual rise in the demand for non-renewable energy sources like oil and natural gas. However, oil and natural gas reservoirs are finite and will eventually be exhausted. Moreover, the extensive utilization of non-renewable resources will worsen the global warming crisis [1,2]. Consequently, effectively leveraging the presently available resources has emerged as a crucial concern. Among the strategies, the utilization of phase-change materials (PCMs) in energy storage or thermal buffering stands out as one of the viable solutions. PCMs are materials that can store and release a significant amount of latent heat during phase-change processes. There are two main types of PCMs: organic and inorganic [3]. Inorganic PCMs include crystalline hydrate salts, fused salts, metals, and alloys. While they exhibit good thermal conductivity during phase changes, their low latent heat and phase separation limit their practical applications [4,5]. Common organic PCMs include alkanes, fatty acids, paraffins, and alcohols. They boast exceptionally high energy storage density and significant application value owing to their solid-liquid phase transition. They offer advantages such as reusability, minimal volume alteration, high energy density, and suitable phase transition temperatures [6,7,8]. Nevertheless, the issue of pollution stemming from leaks arises. Hence, employing polymer monomers for polymerization [9,10,11] to encapsulate PCMs can mitigate leakage concerns while enhancing the contact area with the substrate, thereby improving thermal conductivity efficiency [12,13]. Zhang et al. [9] prepared microencapsulated bio-based n-dodecanol via in-situ polymerization. Yang et al. [10] developed poly(methyl methacrylate)-based phase-change microcapsules for solar energy storage using suspension polymerization. Sun et al. [11] prepared thermal energy storage materials by microencapsulating n-docosane through interfacial polymerization. The polymer materials mainly include polyurea resins [14], urea–formaldehyde resin [15], polymethyl methacrylate [16], styrene [17], and even biodegradable polymers [18]. Lashgari et al. [19] synthesized microcapsules using suspension polymerization. Methyl methacrylate (MMA) and butyl acrylate (BA) were used as the microcapsule shell materials, and hexadecane (HD) was used as the core material. Differential scanning calorimetry (DSC) analysis showed that as the proportion of BA increased, the enthalpy value of the microcapsules decreased. The average particle size was 60–210 μm. Chang et al. [20] prepared microcapsules using in situ polymerization. Urea-formaldehyde was used as the microcapsule shell material, and n-tetradecane was used as the core material. When the amount of emulsifier increased from 0.9% to 1.5%, the average particle size of the microcapsules decreased from 50.9 μm to 37.0 μm. Our previous study [21] developed a PCM microcapsule using mini-suspension polymerization. A silane compound and MMA were copolymerized as the shell, and paraffin was used as the core material. In addition, a thermally conductive inorganic material was added to the shell layer. The results showed that PCM microcapsules can reduce heating and cooling times by approximately 48% and 42%, respectively. When applied to a battery module, the temperature decrease at the center of the module reached 7.3 °C, thereby helping to prolong the service life of the batteries.

Recently, new applications of PCMs have been explored. Combining PCMs with functional materials through microencapsulation can impart unique properties, such as an infrared stealth effect [22,23], magnetic response [24], reversible thermochromic ability [25], and seawater desalination [26]. Zhuang et al. [27] developed a series of magnetic PCM microcapsules with a polymer shell and the PCM (n-octadecane) modified by Fe_3_O_4_ in the core. Additionally, these microcapsules exhibit a high enthalpy of 132 J·g^−1^, demonstrating their suitability for thermal energy storage applications. Lashgari et al. [28] prepared magnetic microcapsules based on a n-hexadecane/Fe_3_O_4_ core and a polymethyl methacrylate (PMMA) shell. Fe_3_O_4_ nanoparticles (NPs) were first modified with oleic acid. Then, they were incorporated into suspension polymerization in the presence of MMA and n-hexadecane. The results showed that the magnetic microcapsules had a wrinkled morphology and a uniform average size of 180 μm. The experimental core content and enthalpy were approximately 24% and 52 J·g^−1^, respectively. Moreover, the thermal degradation temperatures of the magnetic microcapsules were elevated, attributed to the capability of m-Fe_3_O_4_ NPs to absorb heat and improve the thermal resistance of the obtained magnetic PCM microcapsules, thereby retarding n-hexadecane leakage and shell degradation. Researchers have also found that, in addition to their heat storage capability, PCMs exhibit an infrared stealth effect [23,29]. Ke et al. [29] prepared a dual-functional microcapsule with stearic acid as the PCM core and a calcium carbonate shell doped with nano iron. The results showed that the maximum phase transition enthalpy was approximately 161 J·g^−1^, and the infrared emissivity decreased due to the incorporation of nano-iron.

In this study, magnetic phase-change microcapsules with high encapsulation efficiency and enhanced infrared stealth were prepared using mini-suspension polymerization. Paraffin was used as the core material, and a copolymer of MMA and triethoxyvinylsilane was used as the shell material. The triethoxyvinylsilane in the copolymer can enhance the compatibility between the polymer shell and inorganic additives. Additionally, oleic acid-modified Fe_3_O_4_ NPs were added to the shell layer of the microcapsules, and their effects on the particle size, encapsulation efficiency, infrared emissivity, and thermal performance of the microcapsules were investigated.

## 2. Materials and Methods

### 2.1. Materials

Ferric chloride hexahydrate (FeCl_3_·6H_2_O, CAS NO. 10025-77-1), ferrous chloride tetrahydrate (FeCl_2_·4H_2_O, CAS NO. 13478-10-9), oleic acid (CAS NO. 112-80-1), triethoxyvinylsilane (TEVS, CAS NO. 78-08-0), methyl methacrylate (MMA, CAS NO. 80-62-6), ethylene glycol dimethylacrylate (EGDMA, CAS NO. 97-90-5), and polyethylene oxide (PEO, CAS NO. 25322-68-3) with an average molecular weight of 300,000 g/mol were supplied by Alfa Aesar (Haverhill, MA, USA). Benzoyl peroxide (BPO, CAS NO. 94-36-0) was supplied by Acros Organics (Geel, Belgium). Paraffin (CAS NO. 8002-74-2) with a melting point of 43.5–46.4 °C and a density of 0.9 g/cm^3^, polyvinyl alcohol (PVA, CAS NO. 9002-89-5) with a molecular weight of 9000–10,000 g/mol, and polyacrylic acid (PAA, CAS NO. 9003-01-4) with an average molecular weight of 4,000,000 g/mol were purchased from Sigma-Aldrich (Burlington, MA, USA).

### 2.2. Preparation of Oleic Acid-Modified Fe_3_O_4_ (Fe_3_O_4_(m))

First, FeCl_3_·6H_2_O (10.6 g) and FeCl_2_·4H_2_O (4.0 g) were added to a 1000 mL beaker equipped with a nitrogen gas inlet. Meanwhile, 50 mL of ultrapure water was poured into the beaker, and the mixture was stirred at 600 rpm until completely dissolved. Then, 110 mL of 1.5 M NH_4_OH solution was added dropwise to the mixture for 2 h. After all the NH_4_OH solution had been added, stirring was continued for 1 h to obtain Fe_3_O_4_. The reaction for the formation of Fe_3_O_4_ is shown in Equation (1). Next, the Fe_3_O_4_ solution was probe-sonicated for 5 min. Oleic acid was then added and homogenized by sonication for another 5 min. Subsequently, 6 mL of 30% NH_4_OH solution was added and the mixture was stirred at 1000 rpm for 2 h. The pH of the solution was then adjusted to acidic by adding concentrated hydrochloric acid to separate the unreacted oleic acid. The mixture was centrifuged, and the precipitate was rinsed three times with a water/ethanol solution (3:1 by volume). The solid was vacuum dried at 30 °C to obtain oleic acid-modified Fe_3_O_4_ (Fe_3_O_4_(m)) [28].

(1) $${\mathrm{Fe}}^{2+}+2{\mathrm{Fe}}^{3+}+8{\mathrm{OH}}^{-}\to \;{\mathrm{Fe}}_{3}O_{4}+4H_{2}O$$

### 2.3. Preparation of Phase Change Material (PCM) Microcapsules

**Figure 1:**
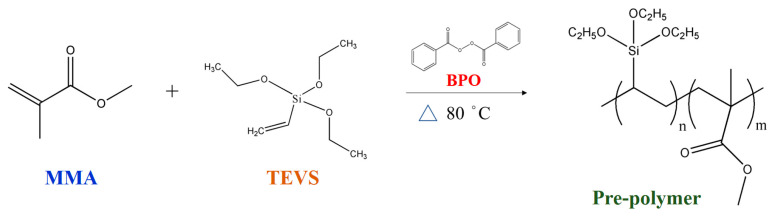
Reaction scheme of the pre-polymer.

**Figure 2:**
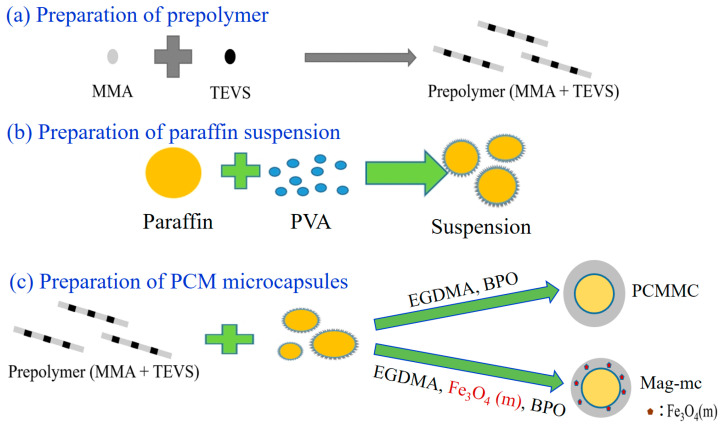
Schematic diagram of microcapsule preparation.

First, 45 g of MMA, 0.045 g of BPO, and 9.0 g of TEVS were prepolymerized under a nitrogen atmosphere in an oil bath at 80 °C for 60 min. The reaction scheme for this prepolymer is shown in Figure 1. Next, 22.5 g of PVA was dissolved in 1500 mL of ultrapure water to prepare a 1.5 wt% PVA aqueous solution before adding 60 g of paraffin. This suspension was placed in a homogenizer and thoroughly mixed at 9800 rpm for 5 min to ensure even dispersion. The prepolymer was added to the prepared suspension and mixed again at 9800 rpm for 5 min. Afterward, 4 g of EDGMA, 5 g of Fe_3_O_4_(m) (with or without), and 0.45 g of BPO were added to the mixture, which was reacted in an oil bath under nitrogen at 80 °C for 24 h with continuous stirring. Finally, the mixture was placed in an ice bath, centrifuged, filtered, and dried to obtain either phase-change material microcapsules (PCMMC) or magnetic phase-change microcapsules (Mag-mc), as depicted in Figure 2.

### 2.4. Preparation of PCM Microcapsule-Containing Films

To a 30 mL ethanol/water (1:1) solution, 0.1 g of PAA and 0.9 g of PEO were added. The mixture was placed in a 60 °C water bath and stirred at 1000 rpm to obtain a clear solution. Then, 0.1 g of PCMMC or Mag-MC was added to the solution and stirred until evenly dispersed. Subsequently, this mixture was poured into a Petri dish and dried in a 40 °C vacuum oven for 2 days to produce F-PCMMC or F-Mag-mc films.

### 2.5. Characterization and Measurement

The infrared spectra of the samples were acquired using a Fourier-transform infrared spectroscopy (FTIR) spectrometer (PerkinElmer Paragon 500, Waltham, MA, USA) with a resolution of 2 cm^−1^. The instrument scanned 50 times across the range of 400 cm^−1^ to 4000 cm^−1^ at room temperature. The crystal structure of as-prepared samples was identified using X-ray diffraction (XRD) with a Bruker D2 PHASER X-ray diffractometer (Billerica, MA, USA) employing CuKα radiation (λ = 1.5405 Å) at 30 kV and 15 mA in the 20 to 80° range, with a scan rate 0.02°(2θ)/min. The magnetic properties of Fe_3_O_4_ and Fe_3_O_4_(m) were characterized using a magnetometer (Quantum Design, MPMS-3, San Diego, CA, USA) in an applied field of up to 10,000 Oe at room temperature. Morphological analysis of the microcapsules was conducted using polarized light microscopy (POM, Carl Zeiss, Oberkochen, Baden-Württemberg, German), and scanning electron microscopy (SEM, JSM-7000F, JOEL, Akishima, Tokyo, Japan). The particle size distribution of the microcapsules was analyzed using an ANALYSETTE 22 NeXT laser analyzer from FRITSCH company (Idar-Oberstein, Germany). The amount of heat absorbed or released during phase transitions was recorded using the differential scanning calorimetry (DSC) with a TA Instruments (New Castle, DE, USA) Q20 instrument (Temperature Accuracy: ±0.1 °C). For DSC analysis, 4–6 mg of microcapsules were placed in an aluminum pan, initially held at −10 °C for 3 min, heated from −10 °C to 200 °C at a rate of 10 °C/min, maintained at 200 °C for 3 min, and then cooled to −10 °C at a rate of −10 °C/min. Thermogravimetric analysis (TGA) was employed to evaluate the thermal stability of the microcapsules using a TA Instruments (New Castle, DE, USA) Q50 instrument (Temperature accuracy: ±0.1 °C, sensitivity: 0.1 μg). For TGA, 4–6 mg of microcapsules were heated to 100 °C at a rate of 10 °C/min, kept at 100 °C for 10 min to remove moisture, and then heated to 600 °C at a rate of 10 °C/min. Infrared thermal images of F-PCMMC and F-Mag-mc were taken with a handheld infrared thermal imager.

## 3. Results and Discussion

### 3.1. FT-IR Analysis

**Figure 3:**
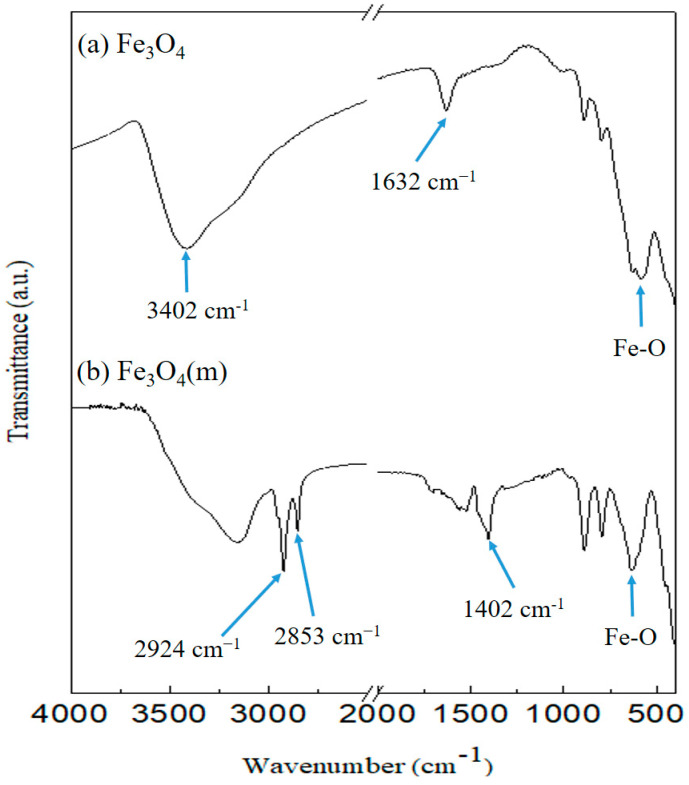
FT-IR spectra of (**a**) pure Fe_3_O_4_ and (**b**) oleic acid-modified Fe_3_O_4_ (Fe_3_O_4_(m)).

The FT-IR spectra of Fe_3_O_4_ and oleic acid-modified Fe_3_O_4_ (Fe_3_O_4_(m)) are presented in Figure 3. The intense peaks between 580 cm^−1^ and 630 cm^−1^ in both Fe_3_O_4_ and Fe_3_O_4_(m) are characteristic of the stretching vibrations associated with the metal-oxygen (Fe–O) bonds. Additionally, the broad peak near 3402 cm^−1^ and the peak at 1632 cm^−1^ correspond to the O–H vibrations of water present in the sample. In the spectrum of Fe_3_O_4_(m), the -CH_2_- symmetric (2853 cm^−1^) and asymmetric (2924 cm^−1^) vibrations of the aliphatic alkyl chains are observed. In addition, the absorption at 1402 cm^−1^ presents a characteristic peak of the COO-Fe bond. It was found that the intensity of the O–H bond near 3402 cm^−1^ decreased significantly, indicating that the carboxylic acid groups of oleic acid had successfully reacted with Fe_3_O_4_ [30,31].

**Figure 4:**
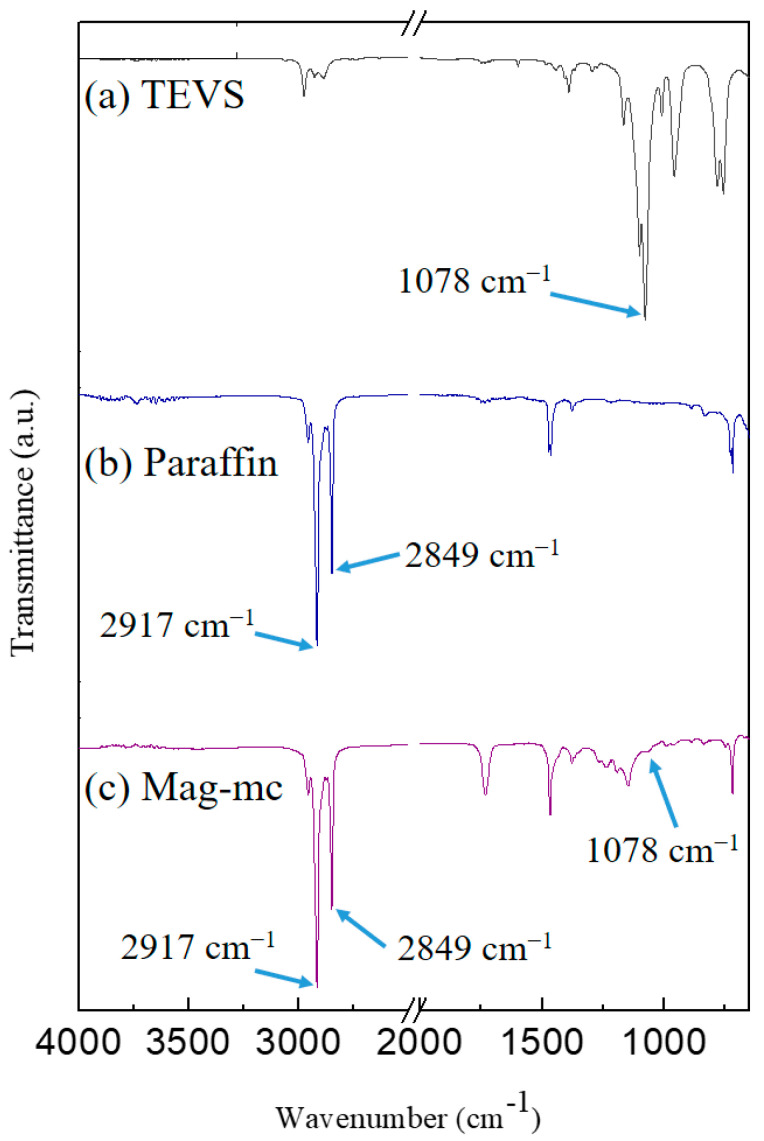
FT-IR spectra of (**a**) triethoxyvinylsilane (TEVS), (**b**) paraffin. and (**c**) Mag-mc.

Figure 4 presents the FT-IR analysis of paraffin, triethoxyvinylsilane (TEVS), and magnetic phase-change microcapsules (Mag-mc). In the paraffin spectrum, two distinctive peaks are observed at 2917 cm^−1^ and 2849 cm^−1^, both of which are indicative of the -CH_2_- functional groups in paraffin. The TEVS spectrum displays a characteristic peak at 1078 cm^−1^ corresponding to the Si-O functional group. Similarly, the Mag-mc spectrum shows prominent peaks at 2917 cm^−1^ and 2849 cm^−1^, suggesting a substantial presence of paraffin in the microcapsules. Additionally, the presence of a characteristic peak at 1078 cm^−1^ for the Si-O functional group confirms the successful synthesis of the magnetic phase-change microcapsules.

### 3.2. X-ray Diffraction Analysis

**Figure 5:**
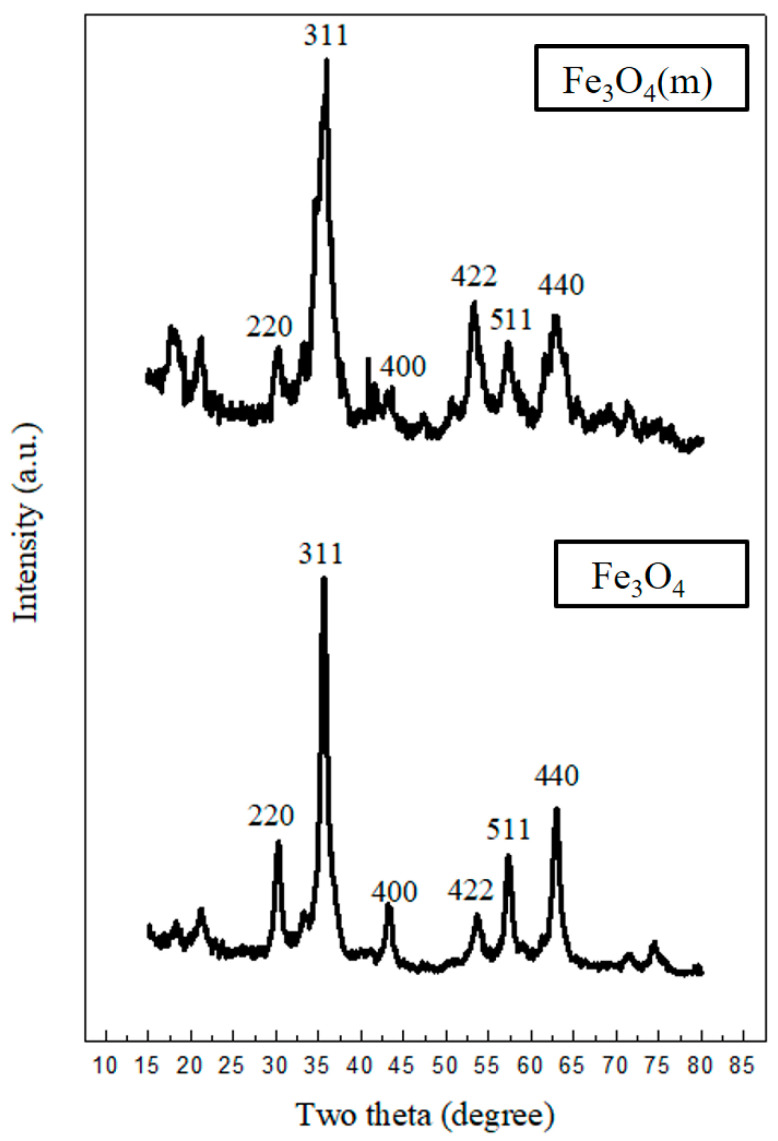
XRD pattern of the Fe_3_O_4_ and oleic acid-modified Fe_3_O_4_ (Fe_3_O_4_(m)).

Figure 5 displays the X-ray diffraction (XRD) pattern of Fe_3_O_4_ and oleic acid-modified Fe_3_O_4_ (Fe_3_O_4_(m)). The sharp diffraction peaks at 2θ = 30.4°, 35.5°, 43.2°, 53.8°, 57.1°, and 63.1° correspond to the crystalline planes (220), (311), (400), (422), (511), and (440), respectively. These peaks confirm that both samples possess an inverse spinel structure, indicative of the successful formation of magnetite [31,32]. The diffraction peaks obtained indicate no impurity phases were detected in the sample. The characteristic peaks for maghemite, hematite, and iron oxide hydroxide at (104), (113), and (130), respectively, were absent [33]. The broadening of the XRD reflections indicates that both Fe_3_O_4_ particles are of nanometric size. The average crystallite sizes of Fe_3_O_4_ and Fe_3_O_4_(m), calculated using the following Debye-Scherrer equation (Equation (2)), are approximately 11.9 nm and 8.3 nm, respectively [34,35]:

(2) $$d=\frac{k\lambda }{\beta \cos\theta }$$

where d is the particle size of magnetite; *k* is a dimensionless shape factor, also known as the Scherrer constant, which is 0.9 for magnetite; λ is the X-ray wavelength; β is the line broadening at half the maximum intensity (FWHM); and θ is the Bragg angle.

Rajan et al. [36] also found that the average crystallite size of citric acid-coated Fe_3_O_4_ (8.4 nm) was smaller than that of the uncoated Fe_3_O_4_ (12.36 nm). This reveals that functionalization is necessary for controlling the size of nanoparticles.

### 3.3. Magnetic Properties

**Figure 6:**
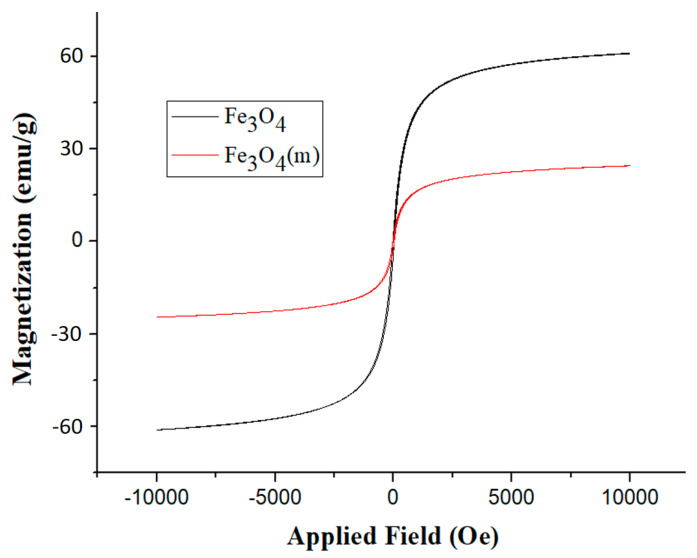
Magnetic hysteresis loops of Fe_3_O_4_ and Fe_3_O_4_(m).

The magnetic properties of Fe_3_O_4_ and Fe_3_O_4_(m) were evaluated using a magnetometer, and the resulting magnetic hysteresis loops are presented in Figure 6. Symmetric hysteresis and saturation magnetization are observed, with both Fe_3_O_4_ and Fe_3_O_4_(m) exhibiting ferrimagnetic behavior. The saturation magnetization (Ms) of Fe_3_O_4_ was 60.93 emu/g, but the Ms of Fe_3_O_4_(m) decreased to 24.57 emu/g. This decrease can be attributed to the surface spin effect on the Fe_3_O_4_ caused by oleic acid-modification, which reduces the saturation magnetization value [31]. Additionally, the ultrasonic irradiation procedure used in preparing Fe_3_O_4_(m) likely caused partial oxidation of Fe_3_O_4_, resulting in reduced magnetization per unit weight of magnetite [37].

### 3.4. Morphology Analysis of Microcapsules

**Figure 7:**
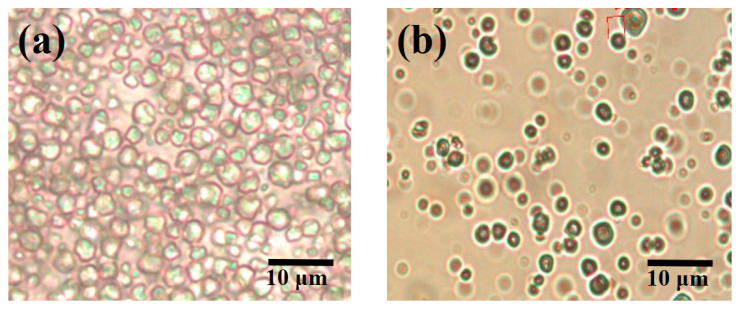
Polarized light microscopy analysis of (**a**) PCMMC and (**b**) Mag-mc.

**Figure 8:**
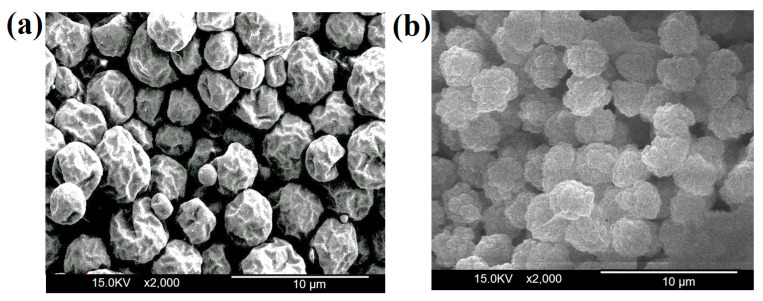
SEM images of (**a**) PCMMC and (**b**) Mag-mc.

According to the polarized light microscopy (POM) analysis (Figure 7), it is evident that the particle size of PCMMC (depicted in Figure 7a) primarily ranges between 3 and 4 μm, with particles being approximately spherical and a wrinkled morphology. Moreover, the Mag-mc microcapsules containing Fe_3_O_4_(m) (shown in Figure 7b) exhibit slightly smaller particle sizes, with a similar spherical shape. Additionally, they may appear darker in color due to the presence of Fe_3_O_4_(m). Scanning electron microscopy (SEM) analysis (Figure 8) similarly demonstrates that the particle size distribution of PCMMC (Figure 8a) primarily falls within the 3 to 4 μm range, showcasing larger variations in size. Conversely, the particle size of Mag-mc microcapsules (Figure 8b) appears more uniform. This indicates that both the particle size and distribution of microcapsules were reduced by the addition of Fe_3_O_4_(m). This may be due to the coexistence of the hydrophobic (alkane) and hydrophilic (COO^−^ and Fe_3_O_4_) ends in oleic acid-modified Fe_3_O_4_ (Fe_3_O_4_(m)). This amphiphilic property plays an important role in promoting the dispersion of paraffin in monomer droplets and polymerizing particles, resulting in Mag-mc microcapsules with smooth outer surfaces, smaller particle sizes, and a narrow size distribution [38].

### 3.5. Particle Size Analysis of Microcapsules

**Figure 9:**
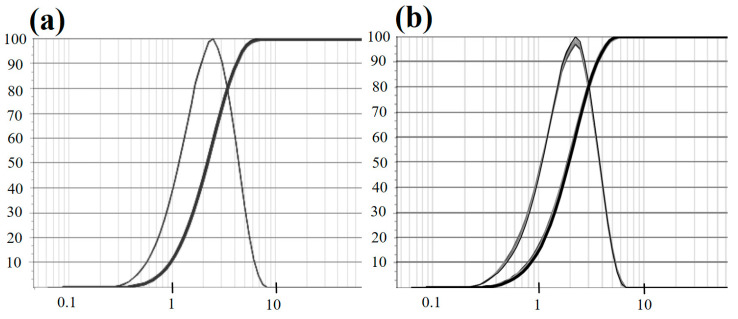
Particle size distribution of (**a**) PCMMC and (**b**) Mag-mc.

Based on the particle size analysis charts of PCMMC and Mag-mc in Figure 9a,b, and the calculations presented in Table 1, Q1, Q2, and Q3 respectively represent the 10%, 50%, and 90% percentiles of the particle size distribution. For PCMMC, the average particle sizes at Q1, Q2, and Q3 are 1.00 μm, 2.20 μm, and 4.20 μm, respectively, while for Mag-mc, the average sizes are 0.86 μm, 1.92 μm, and 3.63 μm. The overall average particle sizes for PCMMC and Mag-mc are 3.48 μm and 2.09 μm, respectively. These results indicate that the particle size distribution ranges for PCMMC and Mag-mc are approximately 1.0–4.2 μm and 0.8–3.6 μm, respectively. Compared to other studies, these results suggest smaller particle sizes and narrower distribution ranges. The incorporation of magnetic materials further reduces both the size and the range of the particle size distribution. This is consistent with the results of the morphology analysis, showing that the amphiphilic property of Fe_3_O_4_(m) promotes the dispersion of paraffin in monomer droplets and polymerizing particles, resulting in smaller particle sizes and a narrow size distribution.

### 3.6. DSC Analysis

**Figure 10:**
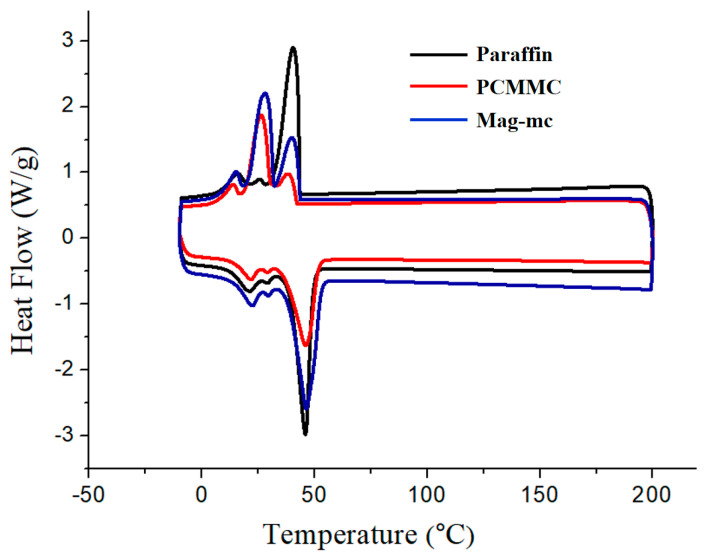
DSC thermograms of paraffin, PCMMC, and Mag-mc.

Figure 10 and Table 2 present the DSC analysis results for paraffin, PCMMC, and Mag-mc. It is found that the melting point of pure paraffin is 45.86 °C with an enthalpy of 119.6 J/g, whereas for PCMMC, the melting point is 46.04 °C with an enthalpy of 95.4 J/g. This suggests that polymer-encapsulated paraffin exhibits a higher melting point due to the lower thermal conductivity of the polymer shell. The encapsulation efficiency calculated using Equation (3) is 79.77% for PCMMC. In addition, the melting point of Mag-mc is 45.26 °C with an enthalpy of 115.0 J/g, and its encapsulation efficiency is calculated to be 96.63%. This indicates that the addition of oleic acid-modified Fe_3_O_4_ (Fe_3_O_4_(m)) in the microcapsules can increase the efficiency of encapsulation, resulting in a 16.86% increase compared to PCMMC. These results are consistent with previous morphological analyses and indicate that the presence of oleic acid on the Fe_3_O_4_(m) surface plays an important role in the encapsulation of PCM within hydrophobic polymer microcapsules. This enhancement enables Mag-mc to possess higher thermal buffering and storage capabilities. Additionally, the presence of Fe_3_O_4_(m) with good thermal conductivity in the shell accelerates heat transfer, leading to a slight decrease in the melting point of the phase-change materials.

(3) $$\mathrm{Encapsulation}\;\mathrm{efficiency}\;(\%)=\frac{\Delta \mathrm{Hm}(\mathrm{Mag}-\mathrm{mc})\;\mathrm{or}\;\Delta \mathrm{Hm}(\mathrm{PCMMC})}{\Delta \mathrm{Hm}(\mathrm{Paraffin})}\times 100\%$$

### 3.7. TGA Analysis

**Figure 11:**
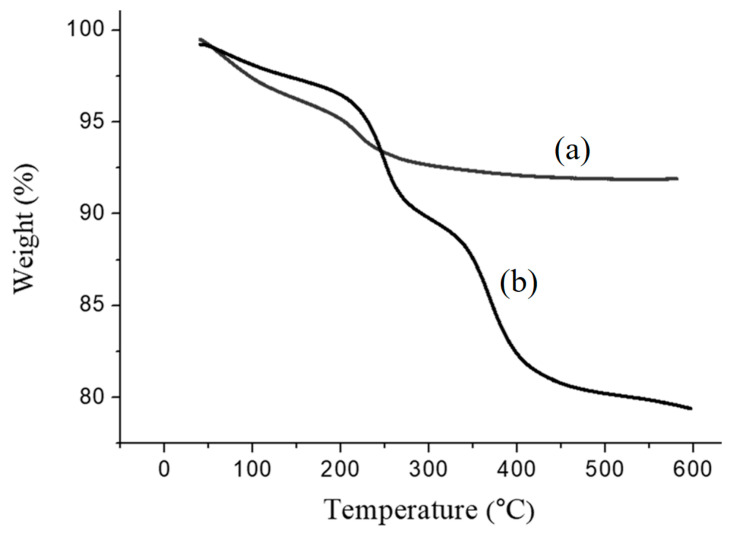
TGA thermograms of (**a**) Fe_3_O_4_ and (**b**) Fe_3_O_4_(m).

**Figure 12:**
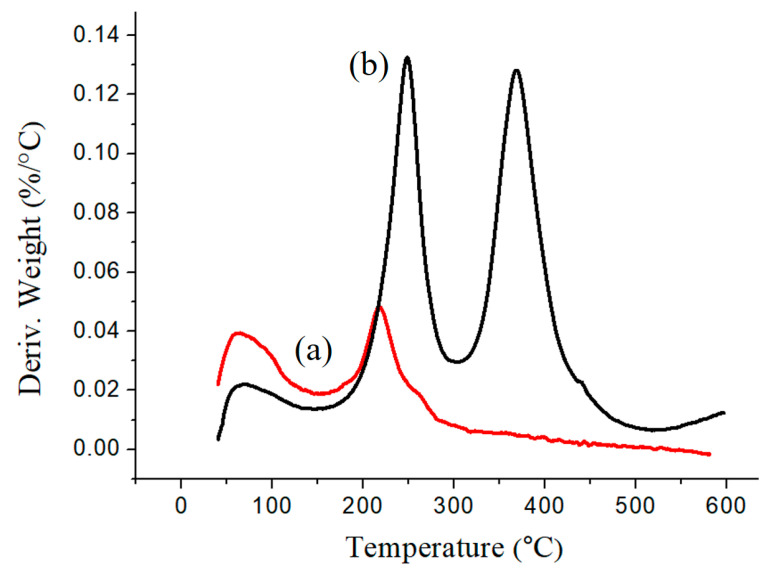
DTG curves of (**a**) Fe_3_O_4_ and (**b**) Fe_3_O_4_(m).

Figure 11 and Figure 12 depict the TGA and DTG analyses of Fe_3_O_4_ and Fe_3_O_4_(m). The thermal decomposition temperatures of Fe_3_O_4_ are 64.4 °C and 217.1 °C, while those of Fe_3_O_4_(m) are 64.8 °C, 247.9 °C, and 368.7 °C. The thermal decomposition temperatures of Fe_3_O_4_ correspond to the evaporation of surface-adsorbed moisture, indicating a char yield of 91.91% [39]. In the case of Fe_3_O_4_(m), the first thermal decomposition temperature of 64.8 °C is also caused by the evaporation of surface-adsorbed moisture. The second thermal decomposition temperature of 247.9 °C arises from the evaporation of surface-adsorbed moisture and the oxidation of oleic acid itself, while the third at 368.7 °C corresponds to the boiling point of oleic acid [40]. The observed char yield is 79.39%. Subtracting the char yield of both samples yields a modified oleic acid content of 12.52%.

**Figure 13:**
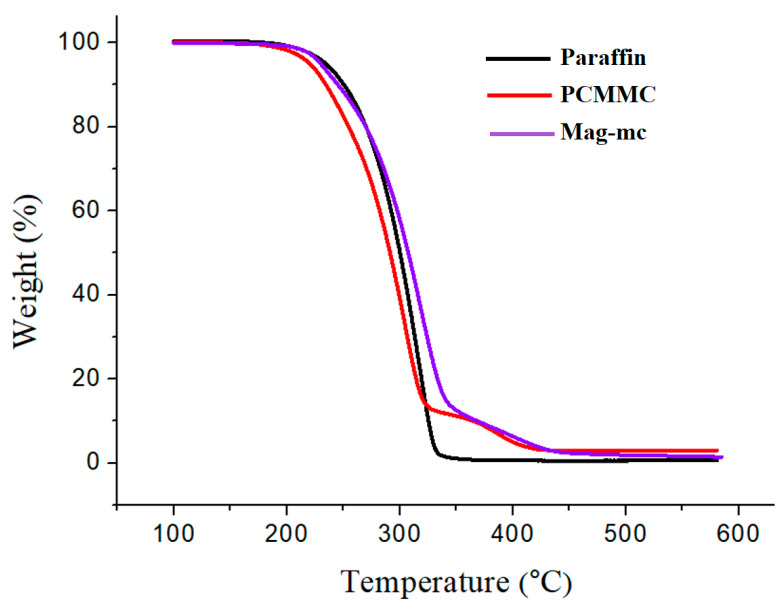
TGA thermograms of paraffin, PCMMC, and Mag-mc.

**Figure 14:**
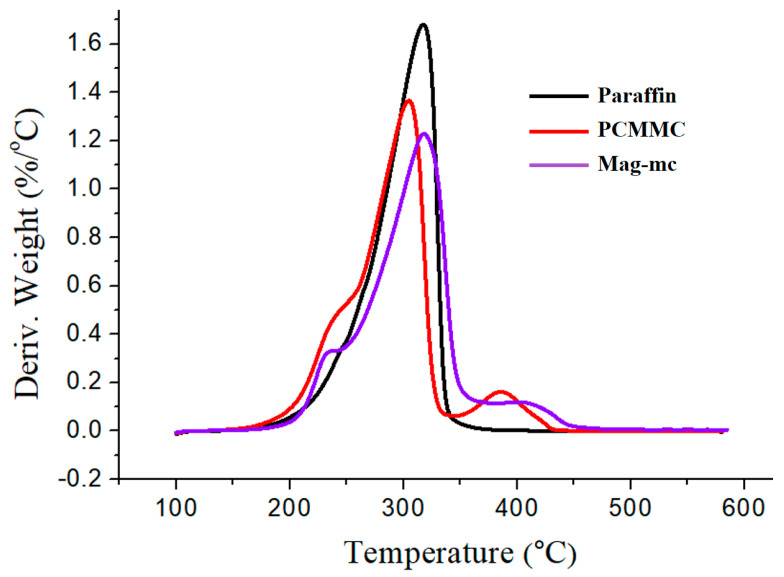
DTG curves of paraffin, PCMMC, and Mag-mc.

Figure 13 and Figure 14, along with Table 3, present the TGA and DTG analyses of paraffin, PCMMC, and Mag-mc. They reveal that the thermal decomposition temperature of paraffin is 317.4 °C. PCMMC exhibits two main thermal decomposition stages: the first stage (304.6 °C) corresponds to the thermal decomposition of PMMA in the microcapsule shell [41] and the paraffin encapsulated within the microcapsule core [42], and the second stage (386.1 °C) corresponds to the thermal decomposition of TEVS in the microcapsule shell [21]. Mag-mc also exhibits two main thermal decomposition stages at 317.9 °C and 413.2 °C, respectively, which are higher than those of PCMMC (304.6 °C and 386.1 °C). This reveals that the thermal stability of the microcapsules is enhanced by the addition of Fe_3_O_4_(m).

### 3.8. Thermal Buffering Capacity Analysis

**Figure 15:**
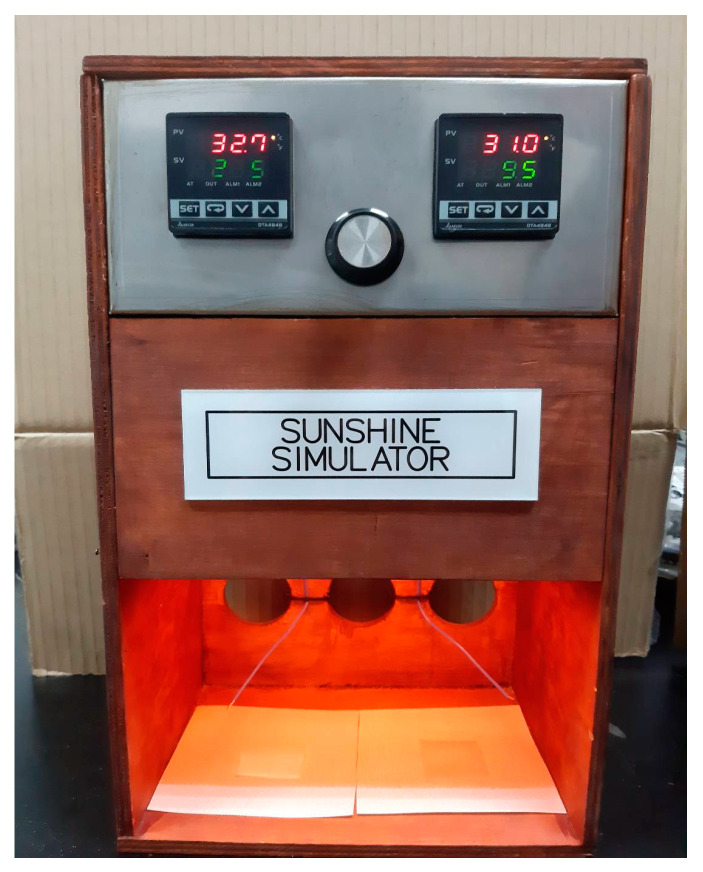
Test process diagram of the thermal buffering capacity analysis of films.

**Figure 16:**
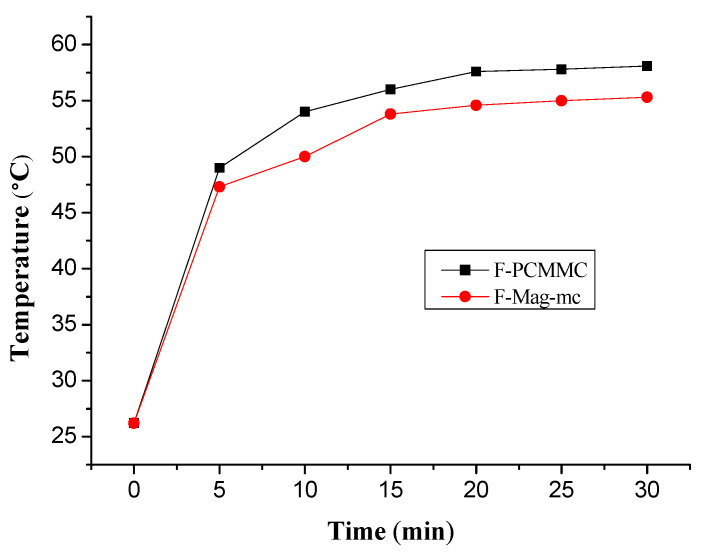
Temperature rise curve of F-PCMMC and F-Mag-mc.

Figure 15 illustrates the thermal buffering capacity analysis of F-PCMMC and F-Mag-mc films. The F-PCMMC and F-Mag-mc films were exposed to simulated sunlight using a sunshine simulator (Burgeon, Taoyuan, Taiwan), and the temperatures of the films were recorded. The analysis results in Figure 16 show that the initial temperature is 26.2 °C. After 5 min of heating, the temperature of F-PCMMC rises from 26.2 °C to 49.0 °C, while F-Mag-mc only rises to 47.3 °C, approximately 1.7 °C lower than F-PCMMC. Moreover, as time progresses, the temperature of F-Mag-mc remains 2–3 °C lower than that of F-PCMMC. This demonstrates that the addition of Mag-mc helps to avoid a rapid temperature increase in the films, achieving the goal of temperature regulation.

### 3.9. Infrared Stealth Effect Analysis

**Figure 17:**
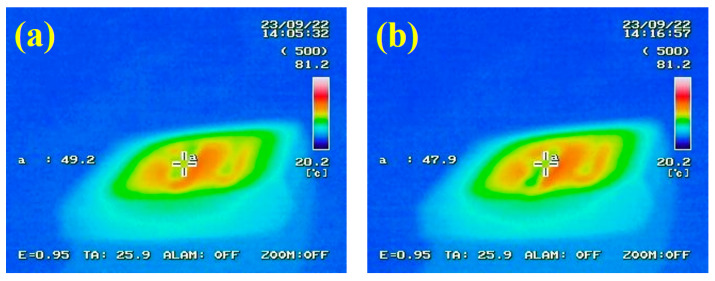
The infrared image of (**a**) F-PCMMC and (**b**) F-Mag-mc on the hot plate heated at 60 °C. for 20 min.

Infrared thermal images were taken by an infrared camera, and the infrared stealth effects of the PCM films were further analyzed. According to the contrast formula of infrared thermality [43] as follows:

(4) $$C=\frac{E_{0}-E_{B}}{E_{B}}$$

where, *C*, E_B_, and E_0_ represent the infrared contrast, the infrared radiation energy of the background, and the infrared radiation energy of the target, respectively. A greater brightness contrast between the sample and the hot plate indicates a more effective infrared stealth effect. Figure 17a,b show the infrared images of F-PCMMC and F-Mag-mc heated on a 60 °C hot plate. The central temperatures of F-PCMMC and F-Mag-mc are 49.2 °C and 47.9 °C, respectively. Subsequently, the infrared emissivity of F-PCMMC was calculated to be 0.77, while the emissivity of F-Mag-mc decreased to 0.72. This indicates that the infrared stealth effect of the PCM films was enhanced by adding Fe_3_O_4_(m) to the shell.

## 4. Conclusions

FT-IR analysis reveals the peaks of oleic acid-modified Fe_3_O_4_ (Fe_3_O_4_(m)) at 2924 cm^−1^ and 2853 cm^−1^, corresponding to the -CH_2_- functional group of oleic acid. Additionally, a characteristic peak of COO-Fe is observed at 1402 cm^−1^, confirming the successful preparation of Fe_3_O_4_(m). Furthermore, the results of XRD patterns confirm that Fe_3_O_4_ and Fe_3_O_4_(m) possess an inverse spinel structure. FT-IR analysis reveals the characteristic peaks of Mag-mc at 2917 cm^−1^, 2849 cm^−1^, and 1078 cm^−1^, corresponding to the -CH_2_- functional group of paraffin and the Si-O functional group, respectively. This confirms the successful synthesis of microcapsules. Moreover, the results reveal that the addition of Fe_3_O_4_(m) in the microcapsules can reduce the particle size of the microcapsule from 3.48 μm to 2.09 μm, and increase the encapsulation efficiency from 79.77% to 96.63%. Nevertheless, this magnetic phase-change microcapsule has an enthalpy as high as 115.0 J/g. This enhancement enables it to possess higher thermal buffering and storage capabilities. Additionally, thermal decomposition analysis showed that the thermal stability of the microcapsules is also enhanced by adding Fe_3_O_4_(m). Thermal buffering capacity analysis showed that the temperature of the Mag-mc-containing film remained 2–3 °C lower than that of the PCMMC-containing film upon exposure to simulated sunlight. Moreover, the infrared emissivity of the Mag-mc-containing film (0.72) was lower than that of the PCMMC-containing film (0.77). These results reveal that the thermal buffering, thermal storage capabilities, and infrared stealth effect of the PCM microcapsules were improved by incorporating Fe_3_O_4_(m) into the shell. The TEVS-modified prepolymer and the amphiphilic properties of oleic acid-modified Fe_3_O_4_ enhance the dispersion and encapsulation of PCM in the microcapsules, leading to improved thermal buffering and storage capacities. In comparison with the results of Lashgari et al. [28], the prepared magnetic microcapsules based on an n-hexadecane/oleic acid-modified Fe_3_O_4_ core and a PMMA shell showed an average size of 180 μm, with a core content and enthalpy of 24% and 52 J·g^−1^, respectively. However, the magnetic microcapsules prepared in this study exhibited a much smaller average size of 2.09 μm, along with a significantly larger core content and enthalpy of 96.63% and 115.0 J·g^−1^. These results indicate that the incorporation of TEVS into the shell layer of the microcapsules enhanced compatibility with the magnetic additive, resulting in smaller size and higher encapsulation efficiency, as well as improved infrared stealth and thermal stability.

## Figures and Tables

**Table 1:** Particle size analysis of PCMMC and Mag-mc.

	PCMMC (μm)	Mag-mc (μm)
Q1	1.00	0.86
Q2	2.20	1.92
Q3	4.20	3.63
Average size	3.48	2.09

**Table 2:** DSC analysis results for paraffin, PCMMC, and Mag-mc.

Sample	T_m_ (°C)	ΔH_m_ (J/g)	Encapsulation Efficiency (%)
Paraffin	45.86	119.6	--
PCMMC	46.04	95.4	79.77
Mag-mc	45.26	115.0	96.63

**Table 3:** TGA analysis results.

Sample	T_d1_ (°C)	T_d2_ (°C)	T_d3_ (°C)	Char Yield (%)
Fe_3_O_4_	64.4	217.143	--	91.91
Fe_3_O_4_(m)	64.8	247.942	368.7	79.39
Paraffin	317.4	--	--	0.69
PCMMC	304.6	386.1	--	3.064
Mag-mc	317.9	413.2	--	1.486

## Data Availability

The original contributions presented in the study are included in the article, further inquiries can be directed to the corresponding author.
